# 668. The clinical effectiveness of Fidaxomicin compared to Vancomycin in the treatment of *Clostridioides difficile* infection in immunocompromised hosts, a single center study

**DOI:** 10.1093/ofid/ofad500.730

**Published:** 2023-11-27

**Authors:** Majd Alsoubani, Jennifer K Chow, Angie Mae Rodday, David Kent, David R Snydman

**Affiliations:** Tufts Medical Center, Boston, Massachusetts; Division of Geographic Medicine and Infectious Diseases, Tufts Medical Center, Boston, MA; Tufts Medical Center, Boston, Massachusetts; Tufts Medical Center, Boston, Massachusetts; Tufts Medical Center, Boston, Massachusetts

## Abstract

**Background:**

Patients with immunocompromising conditions are at increased risk of *C.difficile* infection (CDI) and recurrence. Fidaxomicin reduces the risk of recurrence in immunocompetent hosts. However, there is limited data on fidaxomicin effectiveness in hosts with immunocompromising conditions.

**Methods:**

We retrospectively assessed the treatment of CDI among patients with immunocompromising conditions who were diagnosed between 2011 and 2021 at Tufts Medical Center. Patients were considered to be treated by fidaxomicin or vancomycin if they received ≥72 hours of the agent. Patients less than 18 years, those who did not receive treatment for CDI, and those treated with metronidazole only were excluded. The study outcome was a composite of failure to achieve clinical cure within 72 hours of treatment initiation, relapse within 30 days following completion of initial treatment and death due to CDI. Time to event analysis used a cause specific Cox proportional hazards to compare the rate of the composite outcome in the two groups, accounting for the competing risk of death from other causes. Multiple imputation was used for missing variables but not the outcome.

**Results:**

A total of 238 patients with immunocompromising conditions received vancomycin (n= 38) or fidaxomicin (n= 200) for treatment of CDI. Patients who received vancomycin were significantly more likely to be male (51.5% vs 26.2%, p 0.005) and to have had community acquired infection (31.1% vs 9.5%, p 0.03) compared to fidaxomicin (Table 1). The composite outcome occurred in 48 (24%) patients in the vancomycin group compared to 5 (13.2%) in the fidaxomicin group. In the multivariable model adjusted for sex, number of antecedent antibiotics, antibiotics during treatment, severity and type of immunosuppression, fidaxomicin was associated with 65% reduction in the hazard of the composite outcome compared with vancomycin (HR 0.35, 95% CI 0.12-0.99) (Table 2).

**Figure:**
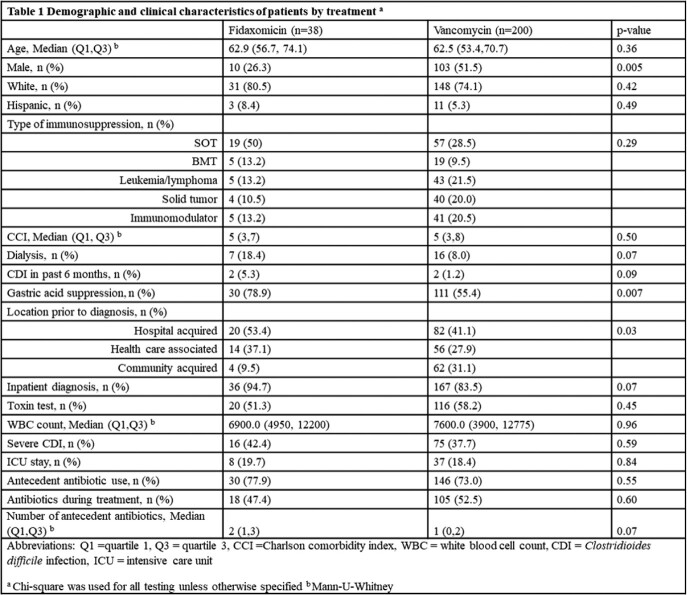

**Figure:**
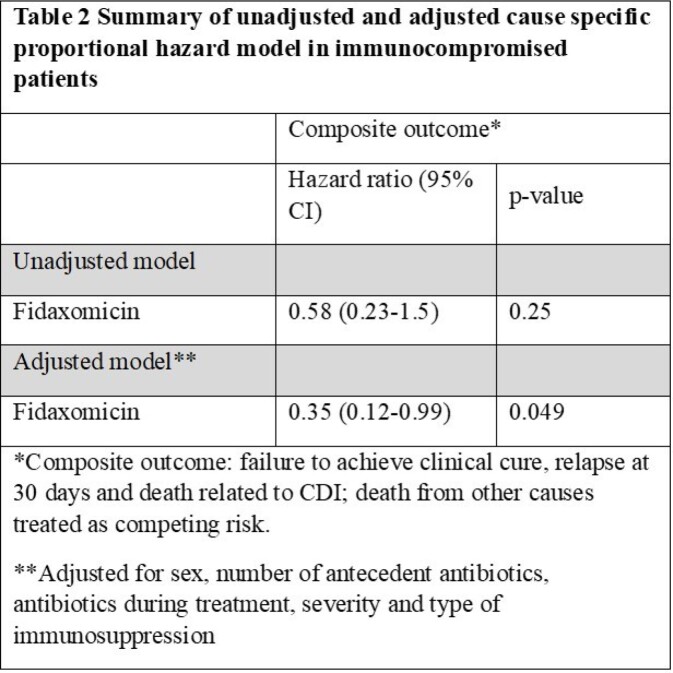

**Conclusion:**

The use of fidaxomicin was more effective than vancomycin in preventing poor CDI outcomes among patients with immunocompromising conditions. The study may have residual confounding by indication and limited generalizability based on a single site. Future studies should confirm our findings.

**Disclosures:**

**Jennifer K. Chow, MD, MS**, Kamada: Grant/Research Support|Merck: Grant/Research Support|Moderna: DSMB **David Kent, MD**, W.L. Gore: Grant/Research Support **David R. Snydman, MD**, Merck: Advisor/Consultant|Merck: Grant/Research Support|Prolacta: Advisor/Consultant|Prolacta: Grant/Research Support|Seres therapeutics: Advisor/Consultant|Seres Therapeutics: Grant/Research Support|Summit Therapeutics: Grant/Research Support

